# Laboratory-acquired dengue virus infection by needlestick injury: a case report, South Korea, 2014

**DOI:** 10.1186/s40557-016-0104-5

**Published:** 2016-04-07

**Authors:** Changhwan Lee, Eun Jung Jang, Donghyok Kwon, Heun Choi, Jung Wan Park, Geun-Ryang Bae

**Affiliations:** Department of Epidemic Intelligence Service, Korea Centers for Disease Control and Prevention, 643 Yeonje-ri, Osong-eup, Cheongju, Heungduk-gu Korea

**Keywords:** Dengue, Laboratories, Needlestick injuries

## Abstract

**Background:**

Dengue fever is one of the most dominant vector-borne diseases, putting approximately 3.9 billion people at risk worldwide. While it is generally vector-borne, other routes of transmission such as needlestick injury are possible. Laboratory workers can be exposed to dengue virus transcutaneously by needlestick injury. This is the first case, to our knowledge, of dengue virus infection by needlestick injury in a laboratory environment. This paper evaluates the risk and related health concerns of laboratory workers exposed to dengue virus.

**Case presentation:**

We evaluated a 30-year-old female laboratory worker exposed to the dengue virus by needlestick injury while conducting virus filtering. During admission, she showed symptoms of fever, nausea, myalgia, and a characteristic maculopapular rash with elevated aspartate aminotransferase (AST) of 235 IU/L and alanine aminotransferase (ALT) of 269 IU/L. She had been diagnosed by a positive nonstructural protein 1 (NS1) antigen (Ag) rapid test one day prior to symptom onset along with positive immunoglobulin M (IgM) enzyme-linked immunosorbent assay (ELISA) on the ninth day of symptom onset. Reverse transcription polymerase chain reaction (RT-PCR), also conducted on the ninth day, was negative. After proper symptomatic treatment, she recovered without any sequelae. As a result of thorough epidemiologic investigation, it was determined that she had tried to recap the needle during the virus filtering procedure and a subsequent needlestick injury occurred.

**Conclusions:**

In the context of health promotion of laboratory workers, we suggest that the laboratory biosafety manual be revised and reinforced, and related prevention measures be implemented. Furthermore, health authorities and health care providers in Korea should be fully informed of proper dengue fever management.

## Background

Dengue fever is a mosquito-borne tropical disease caused by the dengue virus, found worldwide in tropical and subtropical regions such as equatorial Africa, the Americas, and Southeast Asia. The World Health Organization (WHO) reports that about 3.9 billion people in 128 countries worldwide were in danger of dengue fever infection in 2015. It is reported that 390 million people are infected annually, and about 96 million people among them have an apparent infection [1]. In South Korea, dengue fever is a legally designated infectious disease. Its incidence is increasing, with 149 cases in 2012, 252 cases in 2013, and 165 cases in 2014. As of 2014, it is estimated that most dengue infections were brought in from overseas [2]. Dengue fever is usually transmitted by a few species of mosquito within the genus *Aedes*, principally *A. aegypti. Aedes* species that transmit the disease also include *A. albopictus*, *A. polynesiensis*, and *A. scutellaris* [1, 3]. In general, dengue fever is known as a mosquito-borne infectious disease, but it can be transmitted from vertical infection, blood transfusion, organ transplants, and needlestick injuries [4]. Among cases of needlestick injury, health care providers have rarely been reported to have been infected by a syringe needle used for a patient that has dengue virus [4–7]. Biosafety laboratory workers are exposed to diverse pathogenic organisms in the course of their professional work. Exposure routes for them differ according to their job duties and the characteristics of the pathogens they encounter; exposure can be through the respiratory tract, mucous membranes, oral intake, or percutaneous methods. Global laboratory-acquired infections include those from Brucella, Yersinia, and buffalopox, among others [8–11]. A case of dengue fever-related laboratory infection was reported in Australia. In a laboratory, where studies that involved infecting mosquitoes with the dengue virus and disease spread were conducted, one researcher was reported to be infected by the dengue virus that was studied in that laboratory. The exposure route, however, was not clear [12]. This is the first report of an actual non-mosquito vector infection case in South Korea. The authors describe here the first reported dengue fever infection case caused by needlestick injury in a laboratory environment rather than that in a clinical environment, along with a review of the related literature.

## Case presentation

### Patient

Female, 30 years old.

### Chief complaint

Chills, febrile sensation, muscle pain.

### Past medical history or family-related medical history

There was nothing significant to report. No one in her family had ever had dengue fever.

### Social history

None of her coworkers showed similar symptoms or were diagnosed with dengue fever. In the region where she lives, there was no report of any dengue fever case. She had not been recently bitten by any mosquito because it was winter at the time.

### History of overseas travel

A year before she was injured by the syringe needle, she had visited Hong Kong for a short period. She had no other travel history.

### Exposure investigation

**Fig. 1:**
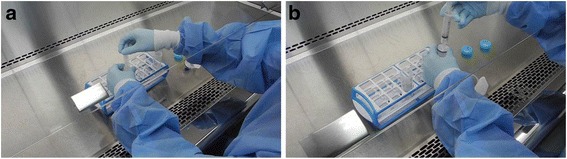
Dengue virus solution filtering procedure. **a** Aspiration. **b** Filtering

**Fig. 2:**
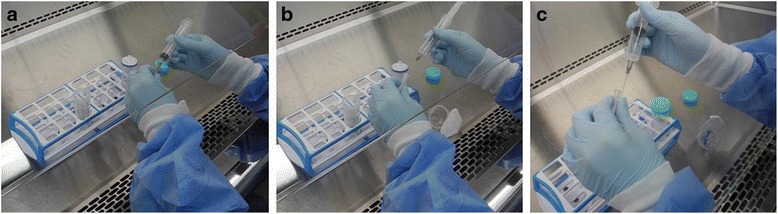
Simulated usage of the needle during the worker’s filtering procedure at the time of injury. **a** Connecting the syringe to the needle. **b** Recapping the needle after use. **c** Needlestick injury

The laboratory where she worked was a biosafety level 2 research facility. When she was injured by the syringe needle, she was in charge of infecting cultured mosquito cells with dengue virus type 2 (DENV-2). During that procedure, she transferred dengue virus solution in a 50 mL conical tube (hereafter referred to as “the tube”) into a 10 mL syringe (hereafter referred to as “the syringe”). Then, she connected the syringe to a disposable filter in order to filter the virus solution (Fig. 1). While she was suctioning the virus solution with the syringe, she removed the needle attached to the syringe. At that moment, the needle cap was not stripped away. However, as she repeated that procedure, the amount of the virus solution contained in the tube decreased. She, therefore, reattached the needle to the syringe and stripped away the cap in order to suction the rest of the virus solution. When she tried to recap the needle to remove it, she was injured (Fig. 2).

### Present medical history

**Fig. 3:**
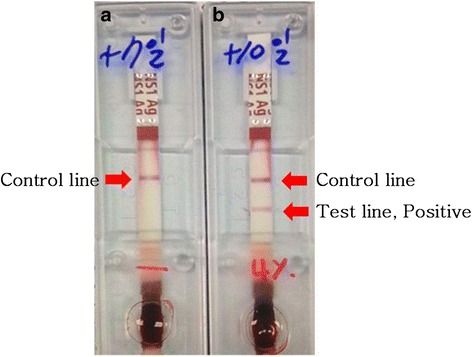
The test results of the NS1 Ag Rapid Test. **a** On the 7th day after the needlestick injury. **b** On the 10th day after the needlestick injury

**Fig. 4:**
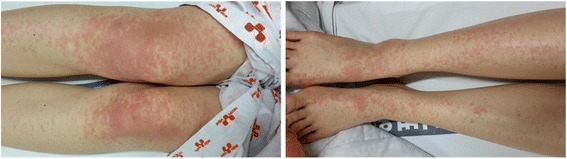
Maculopapular rash of the patient on the third day of admission

The patient washed the wound in running water for 10 min according to laboratory biosafety accident countermeasures and first aid guidelines immediately after she was wounded. She, however, could not disinfect the wound because the necessary materials were not on hand. She went to a nearby hospital, got the wound disinfected, and went home. She kept working in that laboratory unit after the accident. She conducted daily self-tests using the SD BIOLINE Dengue NS1 Ag Rapid Test (Standard Diagnostics Inc., Yongin, South Korea), which is a rapid diagnostic test for dengue virus nonstructural protein 1 (NS1) antigen (Ag). Ten days after she was wounded, a positive test result was confirmed (Fig. 3). Symptoms had started one day before the positive reaction was confirmed. Muscle pains began first. Then, vomiting, chills, and a febrile sensation followed. Two days later, a rash started around both knees (Fig. 4). As symptoms appeared and a positive reaction was confirmed from a diagnostic kit, she went to a university hospital in the city of Gwangju on the third day of the symptoms. However, she did not receive any special examination or treatment. On the next day, the symptoms continued, and she was hospitalized in a community hospital near her home for symptomatic treatment.

### Physical examination

When she was admitted to the hospital, the patient’s blood pressure, pulse, and respiration rate were all within a normal range. Her body temperature measured in the axillary region was 37.3 C, indicating a mild fever. There was a rash around both knees, but physical examination revealed no other abnormalities such as edema or ascites.

### Laboratory examination

The patient’s blood test results when admitted were as follows: complete blood cell count results showed leukocytes of 3,390/mm^3^, hemoglobin of 13.3 g/dL, platelets of 300,000/mm^3^, and hematocrit of 41.0 %, showing slight leukopenia. The blood chemistry examination showed aspartate aminotransferase (AST) of 33 IU/L, alanine aminotransferase (ALT) of 26 IU/L, total protein of 7.7 g/dL, and albumin of 4.3 g/dL, which were within a normal range. Urine examination results were normal. On her sixth day of hospitalization, her blood sample was sent to the Korea National Institutes of Health (KNIH) for confirmatory tests. Immunoglobulin M (IgM) was measured twice using the DENV Detect™ IgM Capture enzyme-linked immunosorbent assay (ELISA) (InBios International, Inc., Seattle, WA, USA) and Panbio® Dengue IgM Capture ELISA (Panbio Diagnostics, Brisbane, Queensland, Australia) once each. Both tests were positive. However, reverse transcription polymerase chain reaction (RT-PCR) was negative (Table 1) [13, 14].

**Table 1:** The results of the diagnostic tests

Tests (Day 17)^a^	Result
DENV Detect™ IgM Capture ELISA^b^	+(ISR 6.5)
Panbio® Dengue IgM Capture ELISA^c^	+(Panbio Units 95.7)
RT-PCR	-

^a^Tests were performed using blood samples collected on the sixth day of hospitalization, which was the seventeenth day after the needlestick injury (Day 0)

^b^InBios International, Inc., USA. Immune status ratio (ISR) values ≥2.84 are considered positive

^c^Panbio Diagnostics, Australia. Panbio Unit values >11.0 are considered positive

### Clinical progress

During hospitalization, she complained of symptoms such as muscle pains, chills, mild fever, nausea, vomiting, and skin rashes accompanied by itchiness. In order to relieve her symptoms, she was treated with acetaminophen, antihistamines, and so on. On the third day of hospitalization, in spite of the ongoing treatments, the muscle pain expanded to her entire body, and the rash kept spreading to other parts of her body. However, on the fifth day of hospitalization, her major symptoms disappeared, except the rash. The rash continuously expanded to her entire body, including her arms, legs, and face. Then, on the seventh day of hospitalization, the rash began to improve.

Blood tests on the third day of hospitalization showed leukocytes of 2,410/mm^3^, showing that leukopenia had worsened. AST and ALT increased to 66 IU/L and 48 IU/L, respectively. On the sixth day of hospitalization, leukocytes were 6,390/mm^3^, showing no leukopenia. However, AST was 235 IU/L and ALT was 269 IU/L, both increasing further. Then she was treated with hepatotonics. On the eighth day of hospitalization, when she was discharged, AST and ALT decreased to 46 IU/L and 145 IU/L, respectively (Table 2). She had neither residual symptoms nor blood parameter abnormalities when she underwent follow-up two weeks later.

**Table 2:** Progress of the laboratory test parameters during hospitalization

	Day 12^a^	Progress		
		Day 14	Day 17	Day 19^b^
White blood cells (/mm^3^)	3,390	2,410	6,390	6,720
Eosinophils (%)	4.4	3.7	0.5	2.1
Hematocrit (%)	41.0	35.0	37.0	37.7
Platelets (/mm^3^)	300,000	194,000	243,000	311,000
AST (IU/L)	33	66	235	46
ALT (IU/L)	26	48	269	145
Total protein (g/dL)	7.7	6.2	7.0	6.7
Albumin (g/dL)	4.3	3.5	3.8	3.6
Total bilirubin (mg/dL)	-	0.5	0.4	-
Direct bilirubin (mg/dL)	-	0.2	-	-

^a^At the time of admission, which was the 12th day after the needlestick injury (Day 0)

^b^At the time of discharge

## Discussion

Exposure to human immunodeficiency virus (HIV), hepatitis B virus (HBV), and hepatitis C virus (HCV) via occupational percutaneous injuries is common globally as well as in South Korea [15, 16]. On the other hand, less than ten reported cases of dengue fever via needlestick injuries have been reported worldwide. Most notably, not a single case like the one reported here, where a laboratory worker experienced the needlestick injury during work, has been reported previously [4]. The exposure investigation revealed that there were no events of exposure to dengue virus such as the vector-borne method by mosquitoes or blood-mediated exposure other than the needlestick injury.

This case has some marked characteristics that differ from those of other reported cases of dengue fever infection by needlestick injury. First, all other reported dengue fever needlestick injury cases have occurred within clinical environments in which dengue fever patients were being treated. However, in this case, a laboratory worker was injured while working in a well-controlled laboratory environment dealing with dengue virus. Second, the laboratory where the infected worker worked was equipped with a rapid diagnosis kit. The worker, therefore, continued to self-test even while symptoms had not yet occurred, and was able to diagnose herself quickly. This is a point worth noting, since it enabled secondary prevention. Thirdly, in this case, proper post-management between the worker and the laboratory was made, so the disease treatment began quickly. The worker, therefore, was able to return to work quickly. She was immediately reassigned to a new task to prevent recurrence. This case, hence, is considered a model case from the standpoints of primary and tertiary prevention.

Here, we will discuss a few issues in three major sections: laboratory biosafety; clinical and epidemiological characteristics of dengue fever infection and job fitness; and general preparedness for dengue fever diagnosis and treatment.

### Laboratory biosafety

Unlike in clinical environments, where procedures are not always done in an orderly manner, we believe that laboratory workers’ exposure to pathogens can be effectively controlled through preventive measures such as having a strict biosafety laboratory facility standard, operation adjustments, substitution of experimental tools, safety devices and equipment, and education to prevent infections by pathogens handled in the laboratory. We, therefore, think that it will be meaningful to find practices in need of improvement among precautionary measures for biosafety laboratory workers by considering the exposure route in this case.

Biosafety laboratories must run their facilities and supply materials according to a strict management standard, and have a safety guide. The procedure that caused the needlestick injury to the worker was as follows: she transferred dengue virus solution from a 50 mL tube into a 10 mL syringe. Then, she connected that syringe with a disposable filter in order to filter the virus solution. She used the syringe by attaching a needle. At the moment she tried to recap the needle, she was injured. There are a few problems to be addressed in this procedure. According to our investigation, when transferring the virus solution to the disposable filter, one could use either a pipette or syringe. When there was a small quantity of virus solution, she used a syringe. At this point, there would have been no problem if she had used the syringe without the needle. However, by attaching the needle to the syringe, she made it possible for the injury to occur. The patient, herself, actually was aware of the risk of a wound while using the needle. However, she thought that the tip of the syringe was too thick to suck in the small amount of the virus solution left in the tube. This is the reason why she used the needle. What’s worse, she recapped the needle after using the syringe. This is the most common cause of needlestick injuries in general. When considering the series of steps during which this accident occurred, we were able to find structural causes other than the patient’s carelessness.

In examining guidelines for safety management of biosafety laboratories, there is a local guideline published by the Korea Centers for Disease Control and Prevention (KCDC) in South Korea. There is also a global manual for laboratory biosafety published by the WHO. In the local guideline, there are restrictions on using syringes, such as limiting the use of sharp material like syringes and replacing them with plastic material if possible [17]. The laboratory biosafety manual published by the WHO, meanwhile, is more specific as follows: Minimize use of syringes and needles. Never recap the needle, and discard it in a separate container for needles only [18]. We think that the manual by the WHO provides more detailed guidelines about using needles than the Korean guidelines. However, we believe that changing expressions in the WHO’s manual; for example, change “minimize using syringes and needles” into “syringes and needles must not be used in a situation where their use is not directly indicated” would be more appropriate in order to remove any potential risk of needlestick injuries. Along with this point, evaluating job fitness about return to work and task allocation for laboratory workers who have been infected must be included in the next revision of the guidelines.

Furthermore, managing and supervising whether these guidelines are properly obeyed or not is also crucial. In this case, the patient was in a situation where she had to use the needle, but there was no separate discarding container for the used needle in the laboratory. Therefore, she had to recap the needle. Furthermore, she washed her wound in running water according to the laboratory’s manual of countermeasures for emergency incidents, but there was no first aid kit that could disinfect the wound in the laboratory. In this case, the fact that she could not disinfect the wound did not greatly affect the disease progression, which was fortunate. We, however, think that taking appropriate action at the beginning of the infection incident is essential to prevent any secondary infection of contaminated wounds. It will, therefore, be vital to supervise whether safety equipment is managed according to safety guidelines.

### Dengue fever and job fitness of laboratory workers

We also need to address the health care of laboratory workers and dengue fever infection in more depth. Dengue virus is a small single-stranded RNA virus, and there are four distinct dengue virus serotypes, from type 1 to type 4. Concurrent or secondary infections of different serotypes are possible. When infected, patients are classified into either non-severe dengue or severe dengue. Non-severe dengue is divided again into two subgroups: those with warning signs and those without warning signs. Warning signs are as follows: abdominal pain or tenderness; persistent vomiting; clinical fluid accumulation; mucosal bleeding; lethargy, restlessness; liver enlargement by more than 2 cm; and an increase in hematocrit concurrent with a rapid decrease in platelet count. Those with warning signs are prone to severe dengue. Criteria for probable dengue fever include fever and two of the following: nausea, vomiting; rash; aches and pains; positive tourniquet test; leucopenia; or any warning signs. Severe dengue is life-threatening and comprises severe plasma leakage leading to shock, severe bleeding, and severe organ involvement [3]. High body mass index (BMI), being a child or female, chronic diseases like asthma or diabetes, high viral load, and concurrent or secondary infection with other serotypes of dengue virus are associated with a greater risk of a severe clinical course [19–28]. Among these risk factors, we will discuss concurrent or secondary dengue virus infection at work and possible high viral load during infection by a needlestick injury.

In this case, there was a needlestick injury, and it then took 10 days until a positive result appeared on the Dengue NS1 Ag Rapid Test. The patient kept working in the same unit using the same virus filtering method described above until the dengue fever symptoms appeared. The operation in the unit she worked for dealt with all four dengue virus serotypes. Therefore, she was exposed to an environment where she could have been infected with some or all of the four dengue virus serotypes during the filtering operations. It has been suggested that if the patient becomes infected by several dengue serotypes concurrently, the case may display a more severe clinical course, though this remains under question [19–23]. Even if the greater potential risk of multiple serotype infection is controversial, we believe it is appropriate to isolate workers who have experienced needlestick injuries from all work handling the dengue virus until a definite diagnosis is made in order to protect them. Moreover, if a concurrent infection from other pathogens such as hepatitis C or malaria occurs, it can worsen the clinical course [29–32]. Thus, allocating workers infected with dengue virus should be done with careful consideration.

It is also important that the potential for secondary dengue virus infection be taken into consideration in terms of the worker’s future job compatibility. If there is a secondary infection by another dengue virus serotype, the risk of a severe case of dengue fever increases. Specifically, when a person who was infected with dengue virus type 1 (DENV-1) is infected secondarily with DENV-2 or dengue virus type 3 (DENV-3), or when a person who was infected with DENV-3 is infected secondarily with DENV-2, it has been reported that the risk of severe dengue fever increases [24, 33]. Further research must be undertaken in order to elucidate the effects of related risk factors since there have been few studies on factors of secondary dengue virus infection that may increase severe dengue fever, such as combinations of serotypes and gaps between primary and secondary infections. In this case, the laboratory reallocated the patient to another unit that did not handle the dengue virus at all when the patient finished dengue fever treatment to prevent secondary dengue virus infection. Essential information, however, was not provided by the health care providers during the treatment or right after its completion. It was fortunate that, in this case, those at the laboratory had sufficient knowledge about dengue fever. A medical evaluation must be made jointly among medical experts, the laboratory and the laboratory workers prior to the worker’s return to work. Furthermore, in non-occupational environments such as traveling to a dengue fever-endemic region, the patients should be aware of and avoid secondary dengue virus infections.

It is known that, in general, about 10 to 20 dengue virus copies are needed for a person to be infected with dengue virus by a mosquito bite. In case of a needlestick injury, however, more virus copies are needed than in the above natural infection course. This number is not precisely known, but the number for HIV infection by needlestick injury is approximately 500. It is, therefore, expected that a similar number of dengue virus copies may cause infection [4]. As discussed above, the number of virus copies required for the needlestick injury would be more than for a mosquito bite infection. A relationship between the number of dengue virus copies at the moment of infection and viral load during the infection has not been clearly determined. Thus, further research is required on whether the viral load during the infection by needlestick injury is higher than that via mosquito bite infection, given that more virus copies are needed for the needlestick injury-mediated infection.

### General preparedness for clinical dengue management in South Korea in question

Besides the matters discussed above, the general preparedness of medical professionals in South Korea for dengue fever should also be addressed. On the third day of symptom onset, the patient went to a university hospital for treatment. The health care providers of the hospital just had the patient return home based on an observation of the relatively mild symptoms of the patient at that moment, without performing essential tests such as physical examinations or blood tests. They should have evaluated the patient’s condition clinically and taken initial actions to observe the clinical course because dengue fever may transform into a fatal case of severe dengue fever without showing any special symptoms at the beginning [3]. On the next day, the patient was hospitalized near her home. Routine blood tests including a complete blood cell count were conducted, but no tests were done for differential diagnosis. Plus, laboratory tests for definite diagnosis of the dengue fever were only performed on the sixth day of hospitalization (the ninth day of symptom onset).

Laboratory tests to diagnose dengue fever include virus isolation using cell culture, nucleic acid detection using RT-PCR, NS1 Ag detection, and serological methods such as IgM or immunoglobulin G (IgG) detection. Viral antigen and nucleic acid methods of detecting the virus can be used when a clinical specimen has been collected during the viremic period, which is one to five days after the symptom onset. After this period, serological methods can be used. Direct virus detection is more reliable than the indirect serological method [3]. To confirm a diagnosis of dengue fever according to the Dengue Control (DENCO) study, the following conditions must be met: PCR is positive; it is positive in virus culture; IgM seroconversion is confirmed in paired serum; IgG seroconversion is confirmed in paired serum; and an IgG titer increase of at least 4-fold is confirmed. Moreover, the probability of infection by dengue fever is highly suggested when IgM is positive in one serum test or the titer of IgG is above 1:1280 [3, 34].

In this case, the serum test was conducted once using a specimen collected on the ninth day of symptom onset and was sent to the testing facility to make the diagnosis, which was too late for direct diagnostic methods. Consequently, test results for RT-PCR were negative and results for IgM were positive. This was not sufficient to diagnose the case as dengue fever with certainty according to the criteria of the DENCO study. However, in this case, the patient conducted self-tests and kept the result of the Dengue NS1 Ag Rapid Test, which showed a positive reaction, and this helped the patient to be diagnosed with dengue fever. The SD BIOLINE Dengue NS1 Ag Rapid Test (Standard Diagnostics Inc., Yongin, South Korea) used by the patient showed 72.4 % sensitivity and 100 % specificity in a study that compared the efficacy of several dengue NS1 Ag rapid tests [35]. Moreover, dengue fever can be suspected clinically aside from laboratory methods of diagnosis. In this case, the patient showed clinical symptoms that fit dengue fever (fever with nausea, vomiting, muscle pains, characteristic maculopapular rash, and leukopenia) along with the serological test and Dengue NS1 Ag Rapid Test, increasing the reliability of the dengue fever diagnosis [24].

The implications of this case for the dengue fever diagnosis process are twofold. First, the reliability of dengue fever diagnosis by domestic health care providers in Korea can be low due to insufficient experience and preparedness. Based on how this case was handled, the health care providers seem to have lacked knowledge of appropriate dengue fever treatment or diagnosis guidelines. Therefore, related institutions and health authorities should provide continuing education and training programs to prepare for an increase in dengue fever patients in the future. Second, the laboratory worker handling the dengue virus used the Dengue NS1 Ag Rapid Test, and this enabled rapid diagnosis and treatment. Although perfect countermeasures that fit with the guidelines were not performed during the diagnosis and treatment process, the patient was motivated to keep visiting medical institutions, allowing for timely diagnosis and treatment, which is very important. When an infection incident happens in a laboratory handling the dengue virus, arranging for a rapid test kit may help protect laboratory workers’ health by enabling timely diagnosis and treatment.

## Conclusion

This case concerned a non-mosquito vector dengue fever infection, where the laboratory worker was infected from a needlestick injury in a laboratory environment. We believe that proper prevention measures, such as revising the guidelines and considering job fitness, must be implemented to promote and protect laboratory workers’ health. Dengue fever is not endemic in South Korea, and general preparedness is relatively low. However, we expect that more patients in countries like South Korea without endemic dengue fever will suffer from dengue fever owing to increasing international travel and global climate change. Therefore, health authorities and health care providers in such countries should be well aware of appropriate dengue fever management.

## Consent

Written informed consent was obtained from the patient for publication of this case report and any accompanying images. A copy of the written consent is available for review by the Editor of this journal.

## Abbreviations

Ag: antigen
ALT: alanine aminotransferase
AST: aspartate aminotransferase
BMI: body mass index
DENCO: dengue control
DENV-1: dengue virus type 1
DENV-2: dengue virus type 2
DENV-3: dengue virus type 3
ELISA: enzyme-linked immunosorbent assay
HBV: hepatitis B virus
HCV: hepatitis C virus
HIV: human immunodeficiency virus
IgG: immunoglobulin G
IgM: immunoglobulin M
KCDC: Korea Centers for Disease Control and Prevention
KNIH: Korea National Institutes of Health
NS1: nonstructural protein 1
RT-PCR: reverse transcription polymerase chain reaction
WHO: World Health Organization
